# Synthesis of 3,4-dihydro-1,8-naphthyridin-2(1*H*)-ones via microwave-activated inverse electron-demand Diels–Alder reactions

**DOI:** 10.3762/bjoc.10.24

**Published:** 2014-01-28

**Authors:** Salah Fadel, Youssef Hajbi, Mostafa Khouili, Said Lazar, Franck Suzenet, Gérald Guillaumet

**Affiliations:** 1 Laboratoire de Chimie Organique et Analytique, Université Sultan Moulay Slimane, FST Béni-Mellal, BP 523, 23000 Béni-Mellal, Morocco; 2 Institut de Chimie Organique & Analytique, Université d’Orléans, UMR-CNRS 7311, BP 6759, 45067 Orléans Cedex 2, France; 3 Laboratoire de Biochimie, Environnement & Agroalimentaire, URAC 36, Université Hassan II, FST Mohammedia, BP 146, 20800 Mohammedia, Morocco

**Keywords:** inverse-electron-demand Diels–Alder reaction, microwave irradiation, naphthyridin-2(1*H*)-ones, Sonogashira cross-coupling, 1,2,4-triazine

## Abstract

Substituted 3,4-dihydro-1,8-naphthyridin-2(1*H*)-ones have been synthesized with the inverse electron-demand Diels–Alder reaction from 1,2,4-triazines bearing an acylamino group with a terminal alkyne side chain. Alkynes were first subjected to the Sonogashira cross-coupling reaction with aryl halides, the product of which then underwent an intramolecular inverse electron-demand Diels–Alder reaction to yield 5-aryl-3,4-dihydro-1,8-naphthyridin-2(1*H*)-ones by an efficient synthetic route.

## Introduction

1,8-Naphthyridine derivatives are an important class of heterocyclic compounds and include many substances of both biological and chemical interest [1–4]. Prevention and treatment of angiogenic disorders and cancers were realized with this class of heterocyclic derivatives [5]. They show anti-allergic [6], anti-inflammatory [7], antibacterial [8] and gastric antisecretory activities [9]. Many other remarkable applications are reported in the literature [10–14], such as the selective inhibition of p38 mitogen-activated protein kinase [15] and the potent inhibition of protein kinase C isozymes [16]. Much attention has been devoted to the synthesis of 1,8-naphthyridin-2(1*H*)-ones because of their acyl-CoA:cholesterol acyltransferase (ACAT) inhibitory activity [17] and their role as phosphodiesterase inhibitors [18–19]. To date, 1,8-naphthyridin-2(1*H*)-ones have been prepared mainly by the Knorr or the Friedländer reaction [20–21]. However, these methods cannot give access to various polysubstituted 1,8-naphthyridin-2-ones. Recently, we reported an efficient method for the synthesis of polysubstituted 2,3-dihydrofuro[2,3-*b*]pyridines and 3,4-dihydro-2*H*-pyrano[2,3-*b*]pyridines from 1,2,4-triazines via an inverse electron-demand Diels–Alder reaction under microwave irradiation [22–24]. The use of 1,2,4-triazines in inverse electron-demand Diels–Alder reactions proved to be an efficient strategy for the construction of various heterocyclic compounds [25–27], such as azacarbazoles [28–33], polycyclic condensed pyrazines [34–35], dihydropyrrolopyridines [36–37], thienopyridines and thiopyranopyridines [38–39], as well as furo- and pyranopyridines [22–24,40–42]. Reactions with microwave irradiation are well-known for their ability to reduce reaction times, increase product yields, and reduce unwanted side reactions compared to conventional heating methods [43–49]. In the continuation of our studies on the synthesis of fused heterocyclic systems we decided to extend this methodology to the synthesis of substituted 3,4-dihydro-1,8-naphthyridin-2(1*H*)-ones.

## Results and Discussion

### Synthesis of 1,7-disubstituted 3,4-dihydro-1,8-naphthyridin-2(1*H*)-ones

#### 3-Methylsulfonyl-5-phenyl-1,2,4-triazine

**Scheme 1:**
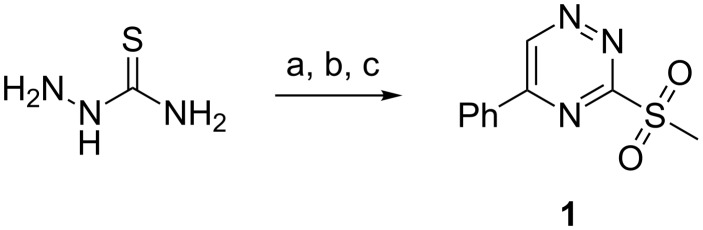
(a) MeI, EtOH, reflux, 3 h (87%); (b) phenylglyoxal, Na_2_CO_3_, H_2_O, 5 °C, 6 h (96%); (c) MCPBA, CH_2_Cl_2_, rt, 4 h (82%).

Our strategy was first based on the 3-methylsulfonyl-1,2,4-triazine **1** (Scheme 1). This key triazine **1** was prepared according to the procedure described by Taylor and Paudler [34,50], i.e., the phenylglyoxal was condensed with the *S*-methylthiosemicarbazide followed by an oxidation reaction with MCPBA.

#### Synthesis of *N*-substituted pent-4-ynamides

**Scheme 2:**
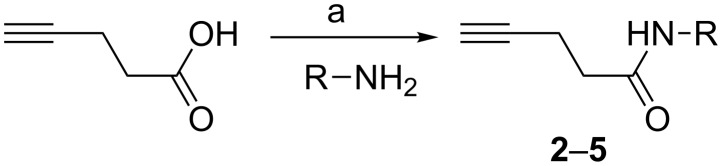
Coupling of pent-4-ynoic acid with different amines. Conditions: (a) EDCI, DMAP, THF, rt, 36 h.

*N*-Alkyl or *N*-aryl-pent-4-ynamides were prepared by amide coupling reactions between pent-4-ynoic acid and various amines in THF in the presence of EDCI and DMAP. The corresponding amides **2–5** were obtained in excellent yields (Scheme 2). The results are shown in Table 1.

**Table 1:** Amide coupling reactions of pent-4-ynoic acid with different amines.

Entry	Amine	Product	Yield (%)^a^

1	butylamine	**2**	96
2	prop-2-en-1-amine [51]	**3**	91
3	isopropylamine	**4**	84
4	aniline [52]	**5**	97

^a^Yield of pure isolated product.

#### Preparation of *N*-substituted *N*-triazinylpent-4-ynamides

**Scheme 3:**
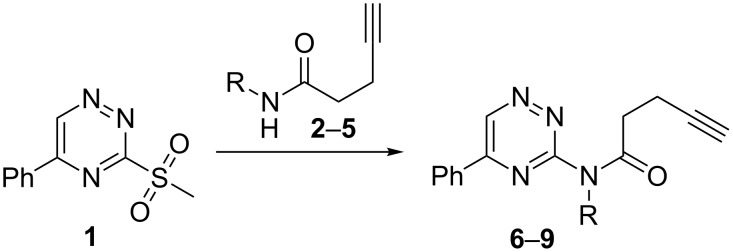
Reaction of triazine **1** with different pent-4-ynamides. Conditions: *n-*BuLi, THF, −30 °C, 2 h.

The nucleophilic substitution of the methylsulfonyl leaving group from **1** by the lithium salt of ynamides **2**–**5** [22–24,53] afforded triazinylpent-4-ynamides **6**–**9** in moderate to good yields (Scheme 3, Table 2).

**Table 2:** Substitution of 1,2,4-triazine **1** by different amides **2–5**.

Entry	R	Product	Yield (%)^a^

1	butyl	**6**	74
2	propenyl	**7**	56
3	isopropyl	**8**	24
4	phenyl	**9**	79

^a^Yield of pure isolated product.

#### Intramolecular inverse electron-demand Diels–Alder reactions

**Scheme 4:**
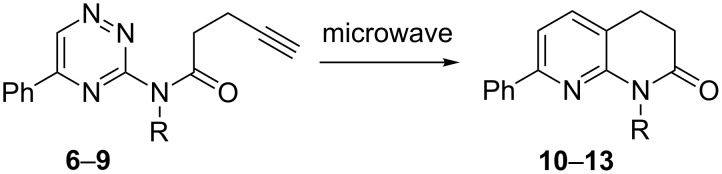
Reaction of triazines **6–9** under microwave irradiation. Conditions: Chorobenzene, 220 °C, 1 h.

With the tethered triazines **6–9** in hand, we were able to study the cycloaddition reaction under microwave heating following the optimal experimental conditions already reported with triazines [22–24]. In chlorobenzene at 220 °C (optimal reaction temperature for six-membered-ring formation), the corresponding cycloadducts **10**–**13** were obtained in high yields (Scheme 4, Table 3).

**Table 3:** Intramolecular inverse electron-demand Diels–Alder reactions under microwave irradiation.

Entry	R	Product	Yield (%)^a^

1	butyl	**10**	97
2	propenyl	**11**	96
3	isopropyl	**12**	93
4	phenyl	**13**	98

^a^Yield of pure isolated product.

We therefore developed an efficient method for the synthesis of 1-substituted 3,4-dihydro-1,8-naphthyridin-2(1*H*)-ones by using 1,2,4-triazine and alkyne tethered together by an amide linker.

#### Synthesis of 1,5,7-trisubstituted-3,4-dihydro-1,8-naphthyridin-2(1*H*)-ones

In order to functionalize the 4-position of the pyridine ring and to extend diversity, we envisaged to evaluate the reactivity of internal alkynes towards the inverse electron-demand Diels–Alder reaction. To reach this goal, we decided to functionalize the alkynes **6–9** employing the Sonogashira cross-coupling reaction.

#### Preparation of aryl-*N*-triazinylpentynamides

**Scheme 5:**
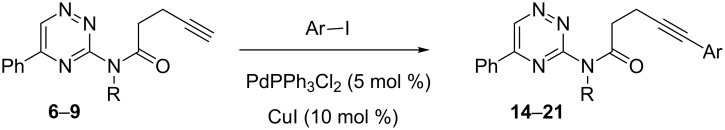
Preparation of aryl-*N*-triazinylpentynamides. Conditions: CuI (10 mol %), Pd(PPh_3_)_2_Cl_2_ (5 mol %), DME, Et_3_N, rt, 3 h.

The terminal alkynes **6–9** were then subjected to a Sonogashira cross-coupling reaction. Thus, treating compounds **6–9** in DME with Pd(PPh_3_)_2_Cl_2_ (5 mol %), CuI, Et_3_N and aryl iodide, gave the cross-coupling products **14–21** in very good yields (Scheme 5). The results are summarized in Table 4.

**Table 4:** Sonogashira cross-coupling reactions from alkynes **6–9**.

Entry	R	Aryl	Product	Yield (%)^a^

1	butyl	2-thienyl	**14**	95
2		4-methoxyphenyl	**15**	95
3	propenyl	2-thienyl	**16**	95
4		4-methoxyphenyl	**17**	89
5	isopropyl	2-thienyl	**18**	91
6		4-methoxyphenyl	**19**	85
7	phenyl	2-thienyl	**20**	86
8		4-methoxyphenyl	**21**	82

^a^Yield of pure isolated product.

#### Intramolecular inverse electron-demand Diels–Alder reactions

**Scheme 6:**
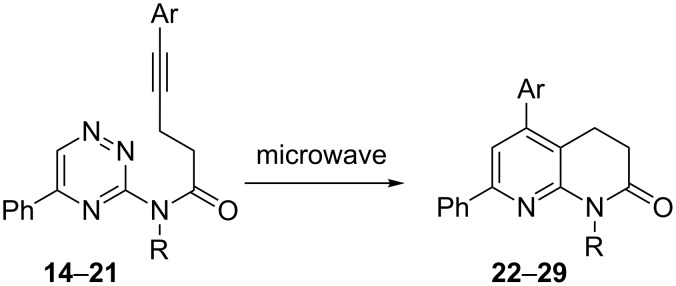
Preparation of 3,4-dihydro-1,8-naphthridin-2(1*H*)-ones. Conditions: Chlorobenzene, 220 °C, 1 h.

Finally, the inverse electron-demand Diels–Alder reaction with tethered triazine **14–21** was carried out under microwave irradiation in a sealed tube at 220 °C (Scheme 6) as previously mentioned [22–24]. The corresponding substituted naphthyridin-2(1*H*)-ones **22–29** were obtained in excellent yields. The results are given in Table 5.

**Table 5:** Intramolecular inverse electron-demand Diels–Alder reactions of substituted alkynes **14–21**.

Entry	R	Ar	Product	Yield (%)^a^

1	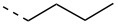	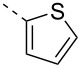	**22**	74
2	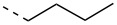	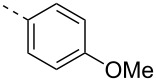	**23**	80
3	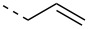	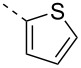	**24**	73
4	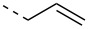	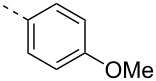	**25**	79
5	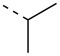	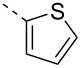	**26**	91
6	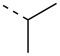	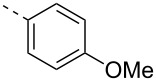	**27**	94
7	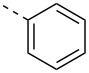	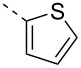	**28**	78
8	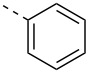	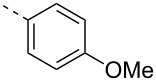	**29**	84

^a^Yield of pure isolated product.

## Conclusion

In this article, we report the successful application of a new synthesis strategy leading to 1-substituted 3,4-dihydro-1,8-naphthyridin-2(1*H*)-ones by inverse electron-demand Diels–Alder reactions under microwave activation. We also synthesized 5-substituted 3,4-dihydro-1,8-naphthyridin-2(1*H*)-ones via the Sonogashira cross-coupling reaction followed by intramolecular inverse electron-demand Diels–Alder reactions. The developed approaches allow a high diversity of substituents on the bicyclic scaffold.

## Supporting Information

File 1 Experimental section.
