# Supplementary material for: Synthesis of 3,4-dihydro-1,8-naphthyridin-2(1H)-ones via microwave-activated inverse electron-demand Diels–Alder reactions
Source: Beilstein J Org Chem. 2014 Jan 28;10:282–6. doi: 10.3762/bjoc.10.24 (PMC3943289; doi:10.3762/bjoc.10.24)
Supplement: File 1 — Experimental section. [file Beilstein_J_Org_Chem-10-282-s001.pdf]

Supporting Information

for

Synthesis of 3,4-dihydro-1,8-naphthyridin-2(1*H*)-ones via microwave-activated inverse electron-demand Diels–Alder reactions

Salah Fadel[1,2], Youssef Hajbi[1,2], Mostafa Khouili[1], Said Lazar[3], Franck Suzenet[*2] and Gérald Guillaumet [*2]

Address: [1]Laboratoire de Chimie Organique et Analytique, Université Sultan Moulay Slimane, FST Béni-Mellal, BP 523, 23000 Béni-Mellal, Morocco, [2]Institut de Chimie Organique & Analytique, Université d'Orléans, UMR-CNRS 7311, BP 6759, 45067 Orléans Cedex 2, France and [3]Laboratoire de Biochimie, Environnement & Agroalimentaire, URAC 36, Université Hassan II, FST Mohammedia, BP 146, 20800 Mohammedia, Morocco

Email: Gérald Guillaumet - gerald.guillaumet@univ-orleans.fr; Franck Suzenet - franck.suzenet@univ-orleans.fr

*Corresponding author

Experimental section

Materials:

Microwave-assisted reactions were carried out in a Biotage Initiator microwave synthesis instrument. Caution, the microwave apparatus has to be equipped with a safety pressure shutoff. Experiments carried out on 0.3 mmol scale of triazine in a 2–5 mL vial can generate 3–6 bar of pressure. ^{1}H NMR and ^{13}C NMR were recorded on a Bruker Avance DPX250 spectrometer (250.19 MHz ^{1}H, 62.89 MHz ^{13}C) using tetramethylsilane as the internal standard, multiplicities were determined by the DEPT 135 sequence, chemical shifts were reported in parts per million (ppm, δ units). Coupling constants were reported in units of hertz (Hz), if applicable. Infrared (IR) spectra were obtained on a Perkin-Elmer Paragon 1000 PC FTIR spectrometer. Infrared spectra were recorded using NaCl films or KBr pellets. Low-resolution mass spectra (MS) were recorded on a Perkin-Elmer SCIEX API 3000 spectrometer. Melting points were determined in

open capillary tubes and are uncorrected. Flash chromatography was performed on silica gel 60 (40–63 mesh). Thin layer chromatography (TLC) was carried out on Merck silica gel 60F_{254} precoated plates. Visualization was made with ultraviolet light. Reactions requiring anhydrous conditions were performed under argon. Ethylene glycol dimethyl ether, and tetrahydrofuran were freshly distilled from sodium/benzophenone under argon prior to use. Dichloromethane was distilled from calcium hydride under argon prior to use. Chemical products were obtained from Aldrich and Acros.

General procedure for the synthesis of *N*-substituted pent-4-ynamides (2–5)

To a stirred solution of 1-ethyl-3-(3-dimethylaminopropyl)carbodiimide (EDCI) (235 mg, 1.22 mmol, 1.2 equiv) in dry THF (8 mL) under a nitrogen atmosphere, was added pent-4-ynoic acid (100 mg, 1.02 mmol, 1 equiv), DMAP (25 mg, 0.20 mmol, 0.2 equiv) and the appropriated amine (1.12 mmol, 1.1 equiv). The resulting mixture was stirred at room temperature and monitored by TLC. After complete conversion of the starting material, the mixture was diluted with CH_2Cl_2 and washed with brine. The organic layer was separated, dried over $MgSO_4$ and concentrated under vacuo. The resulting residue was purified by silica gel column chromatography (eluant: PE/EtOAc, 6:4) to give the corresponding ynamides **2–5**.

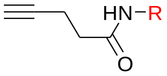

2 - 5

***N*-Butylpent-4-ynamide (2).** Yield 96%, as a white solid; mp 50-52°C; IR (KBr) cm^{-1}: 3303, 2112, 1631; ^{1}H NMR (250 MHz, $CDCl_3$) δ 5.62 (s, 1H), 3.27 (dt, *J* = 6.9 Hz, *J'* = 6.0 Hz, 2H), 2.54 (dt, *J* = 6.6 Hz, *J'* = 2.6 Hz, 2H), 2.38 (t, *J* = 6.7 Hz, 2H), 2.00 (t, *J* = 2.6 Hz, 1H), 1.44-1.53 (m, 2H), 1.33-1.43 (m, 2H), 0.93 (t, *J* = 7.2 Hz, 3H); ^{13}C NMR (62.5 MHz, $CDCl_3$) δ 171.0 (C), 83.2 (C), 69.4 (CH), 39.5 (CH_2), 35.6 (CH_2), 31.8 (CH_2), 20.2 (CH_2), 15.1 (CH_2), 13.9 (CH_3); *m/z* (M+1) = 154.

***N*-Allylpent-4-ynamide (3).** Yield 91%, as a white solid; mp 52-54°C(46-48°C Lit. [1]); IR (KBr) cm^{-1}: 3302, 2110, 1628; ^{1}H NMR (250 MHz, $CDCl_3$) δ 5.76-5.86 (m, 2H), 5.20 (dd, *J* = 17.3 Hz, *J'* = 1.6 Hz, 1H), 5.14 (dd, *J* = 10.3 Hz, *J'* = 1.6 Hz, 1H), 3.87-3.93 (m, 2H), 2.51-2.58 (m, 2H), 2.39-2.45 (m, 2H), 2.00

[1] Pardo, L. M.; Tellitu, I.; Domínguez, E., *Tetrahedron* **2012**, *68*, 3692

(t, J = 2.6 Hz, 1H); ^{13}C NMR (62.5 MHz, $CDCl_3$) δ 170.9 (C), 134.2 (CH), 116.6 (CH_2), 83.1 (C), 69.5 (CH), 42.1 (CH_2), 35.5 (CH_2), 15.0 (CH_2); *m/z* (M+1) = 138.

***N*-Isopropylpent-4-ynamide (4).** Yield 84%, as a white solid; mp 74-76°C; IR (KBr) cm^{-1}: 3303, 2111, 1631; ^{1}H NMR (250 MHz, $CDCl_3$) δ 5.58 (s, 1H), 4.00-4.14 (m, 1H), 2.46-2.54 (m, 2H), 2.33 (t, J = 6.9 Hz, 2H), 1.98 (t, J = 2.6 Hz, 1H), 1.13 (d, J = 6.6 Hz, 6H); ^{13}C NMR (62.5 MHz, $CDCl_3$) δ 170.1 (C), 83.1 (C), 69.4 (CH), 41.6 (CH), 35.6 (CH_2), 22.9 ($2CH_3$), 15.1 (CH_2); *m/z* (M+1) = 140.

***N*-Phenylpent-4-ynamide (5).** Yield 97%, as a white solid; mp 122-124°C (128-129°C Lit. [2]); IR (KBr) cm^{-1}: 3275, 2110, 1665; ^{1}H NMR (250 MHz, $CDCl_3$) δ 7.51 (d, J = 7.9 Hz, 2H), 7.41 (s, 1H), 7.32 (t, J = 7.8 Hz, 2H), 7.11 (t, J = 7.5 Hz, 1H), 2.53-2.68 (m, 4H), 2.06 (t, J = 2.5 Hz, 1H); ^{13}C NMR (62.5 MHz, $CDCl_3$) δ 169.1 (C), 137.6 (C), 129.0 (2CH), 124.4 (CH), 119.8 (2CH), 82.7 (C), 69.7 (CH), 36.3 (CH_2), 14.8 (CH_2); *m/z* (M+1) = 174.

General procedure for the synthesis of *N*-substituted *N*-(5-phenyl-1,2,4-triazin-3-yl)pent-4-ynamides (6–9)

To a stirred solution of **2–5** (0.90 mmol, 1 equiv) in anhydrous THF (2 mL) under a nitrogen atmosphere was added dropwise a solution of *n*-BuLi in hexane (0.5 mL, 1.6 M) at −30 °C. After 30 min, a solution of triazine **1** (0.90 mmol, 1 equiv) in dry THF (1.5 mL) was added and the mixture was stirred at −30 °C. After 2 hours the reaction was quenched with water, allowed to reach slowly room temperature and extracted with EtOAc. The combined organic extracts were dried over $MgSO_4$, evaporated and purified by column chromatography (eluent; PE/EtOAc, 8:2).

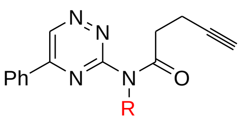

6 - 9

***N*-Butyl-*N*-(5-phenyl-1,2,4-triazin-3-yl)pent-4-ynamide (6).** Yield 74%, as a yellow oil; IR (NaCl) cm^{-1}: 1683; ^{1}H NMR (250 MHz, $CDCl_3$) δ 9.47 (s, 1H), 8.15 (dd, J = 8.0 Hz, J' = 1.5 Hz, 2H), 7.54-7.64 (m, 3H), 4.21 (t, J = 7.5 Hz, 2H), 3.12 (t, J = 7.5 Hz, 2H), 2.62 (dt, J = 7.4 Hz, J' = 2.6 Hz, 2H), 1.92 (t, J = 2.6

[2] Kutschy, P., Kristian, P., Dzurilla, M., Koscik, D., Nadaskay, R., *Collect. Czech. Chem. Commun.***1987**, *52*, 995-1005

Hz, 1H), 1.64-1.74 (m, 2H), 1.32-1.41 (m, 2H), 0.93 (t, *J* = 7.2 Hz, 3H); ^{13}C NMR (62.5 MHz, $CDCl_3$) δ 172.9 (C), 161.7 (C), 155.9 (C), 142.0 (CH), 133.1 (CH), 133.0 (C), 129.6 (2CH), 127.8 (2CH), 83.4 (C), 68.8 (CH), 46.6 (CH_2), 36.8 (CH_2), 30.6 (CH_2), 20.3 (CH_2), 15.2 (CH_2), 13.9 (CH_3); *m/z* (M+1) = 309.

***N*-Allyl-*N*-(5-phenyl-1,2,4-triazin-3-yl)pent-4-ynamide (7).** Yield 56%, as a yellow solid; mp 94-96°C; IR (KBr) cm^{-1}: 1683; ^{1}H NMR (250 MHz, $CDCl_3$) δ 9.48 (s, 1H), 8.16 (dd, *J* = 8.0 Hz, *J'* = 1.6 Hz, 2H), 7.55-7.67 (m, 3H), 5.89-6.05 (m, 1H), 5.24 (dd, *J* = 17.3 Hz, *J'* = 1.6 Hz, 1H), 5.13 (dd, *J* = 10.1 Hz, *J'* = 1.6 Hz, 1H), 4.86-4.89 (m, 2H), 3.20 (t, *J* = 7.4 Hz, 2H), 2.66 (dt, *J* = 7.4 Hz, *J'* = 2.6 HZ, 2H), 1.93 (t, *J* = 2.6 Hz, 1H); ^{13}C NMR (62.5 MHz, $CDCl_3$) δ 172.7 (C), 161.4 (C), 156.0 (C), 142.2 (CH), 133.2 (CH), 133.1 (CH), 133.0 (C), 129.7 (2CH), 127.9 (2CH), 117.5 (CH_2), 83.4 (C), 68.9 (CH), 48.5 (CH_2), 36.9 (CH_2), 15.1 (CH_2); *m/z* (M+1) = 293.

***N*-Isopropyl-*N*-(5-phenyl-1,2,4-triazin-3-yl)pent-4-ynamide (8).** Yield 24%, as a yellow oil; IR (NaCl) cm^{-1}: 1670; ^{1}H NMR (250 MHz, $CDCl_3$) δ 9.59 (s, 1H), 8.19 (dd, *J* = 8.5 Hz, *J'* = 1.5 Hz, 2H), 7.55-7.65 (m, 3H), 5.05-5.17 (m, 1H), 2.53-2.68 (m, 4H), 1.88 (t, *J* = 2.5 Hz, 1H), 1.36 (d, *J* = 6.9 Hz, 6H); ^{13}C NMR (62.5 MHz, $CDCl_3$) δ 171.3 (C), 161.6 (C), 156.4 (C), 143.5 (CH), 133.3 (CH), 132.7 (C), 129.8 (2CH), 127.9 (2CH), 83.3 (C), 68.8 (CH), 49.3 (CH), 35.6 (CH_2), 21.0 ($2CH_3$), 14.9 (CH_2); *m/z* (M+1) = 295.

***N*-Phenyl-*N*-(5-phenyl-1,2,4-triazin-3-yl)pent-4-ynamide (9).** Yield 79%, as a yellow solid; mp 124-126°C; IR (KBr) cm^{-1}: 1692; ^{1}H NMR (250 MHz, $CDCl_3$) δ 9.50 (s, 1H), 8.04-8.9 (m, 2H), 7.37-7.60 (m, 8H), 2.96 (t, *J* = 8.3 Hz, 2H), 2.62-2.69 (m, 2H), 1.96 (t, *J* = 2.6 Hz, 1H); ^{13}C NMR (62.5 MHz, $CDCl_3$) δ 172.6 (C), 162.2 (C), 156.6 (C), 143.0 (CH), 140.2 (C), 133.1 (CH), 132.8 (C), 129.8 (2CH), 129.6 (2CH), 129.0 (2CH), 128.5 (CH), 128.0 (2CH), 83.1 (C), 69.1 (CH), 35.7 (CH_2), 14.8 (CH_2); *m/z* (M+1) = 329.

General procedure for the Sonogashira cross-coupling reaction

A solution of 2-iodothiophene or 1-iodo-4-methoxybenzene (0.28 mmol, 1 equiv) in DME (2 mL) was treated with the appropriate triazines **6–9** (0.30 mmol, 1.1 equiv) and triethylamine (3 mL). After 5 min, CuI (0.028 mmol, 10%) and $Pd(PPh_3)_2Cl_2$ (0.014 mmol, 5%) were added. The mixture was then stirred vigorously at room temperature and monitored by TLC. After 3 hours the mixture was diluted with EtOAc and filtered through celite. The filtrate was washed with brine and dried over $MgSO_4$, evaporated and

purified by column chromatography (eluent; PE/EtOAc, 7.5:2.5) to give the corresponding compounds **14–21**.

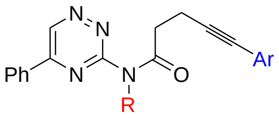

14 - 21

***N*-Butyl-*N*-(5-phenyl-1,2,4-triazin-3-yl)-5-(thiophen-2-yl)pent-4-ynamide (14).** Yield 95%, as a yellow oil; IR (NaCl) cm^{-1}: 1674; ^{1}H NMR (250 MHz, $CDCl_3$) δ 9.47 (s, 1H), 8.16 (d, J = 6.3 Hz, 2H), 7.54-7.63 (m, 3H), 7.14 (d, J = 5.1 Hz, 1H), 7.07 (d, J = 3.8 Hz, 1H), 6.89 (dd, J = 5.0 Hz, J' = 3.8 Hz, 1H), 4.24 (t, J = 7.5 Hz, 2H), 3.21 (t, J = 7.5 Hz, 2H), 2.88 (t, J = 7.5 Hz, 2H), 1.67-1.75 (m, 2H), 1.33-1.44 (m, 2H), 0.92 (t, J = 7.2 Hz, 3H); ^{13}C NMR (62.5 MHz, $CDCl_3$) δ 173.0 (C), 161.8 (C), 156.0 (C), 142.0 (CH), 133.1 (CH), 131.3 (CH), 129.7 (2CH), 129.4 (C), 127.9 (2CH), 126.8 (CH), 126.2 (CH), 123.9 (C), 93.0 (C), 74.3 (C), 46.7 (CH_2), 36.9 (CH_2), 30.6 (CH_2), 20.3 (CH_2), 16.5 (CH_2), 14.0 (CH_3); *m/z* (M+1) = 391.

***N*-Butyl-*N*-(5-phenyl-1,2,4-triazin-3-yl)-5-(4-methoxyphenyl)pent-4-ynamide (15).** Yield 95%, as a maroon solid; mp 72-74°C; IR (KBr) cm^{-1}: 1674; ^{1}H NMR (250 MHz, $CDCl_3$) δ 9.48 (s, 1H), 8.17 (dd, J = 7.8 Hz, J' = 1.3 Hz, 2H), 7.54-7.64 (m, 3H), 7.27 (dd, J = 6.9 Hz, J' = 1.9 Hz, 2H), 6.77 (dd, J = 6.9 Hz, J' = 2.2 Hz, 2H), 4.25 (t, J = 7.5 Hz, 2H), 3.78 (s, 3H), 3.22 (t, J = 7.8 Hz, 2H), 2.85 (t, J = 7.8 Hz, 2H), 1.66-1.79 (m, 2H), 1.35-1.45 (m, 2H), 0.94 (t, J = 7.2 Hz, 3H); ^{13}C NMR (62.5 MHz, $CDCl_3$) δ 173.2 (C), 161.8 (C), 159.1 (C), 155.9 (C), 142.0 (CH), 133.0 (2CH), 132.9 (CH), 129.6 (2CH), 129.6 (C), 127.8 (2CH), 115.8 (C), 113.8 (2CH), 87.3 (C), 80.8 (C), 55.3 (CH_3), 46.6 (CH_2), 37.1 (CH_2), 30.6 (CH_2), 20.3 (CH_2), 16.3 (CH_2), 13.9 (CH_3); *m/z* (M+1) = 415.

***N*-Allyl-*N*-(5-phenyl-1,2,4-triazin-3-yl)-5-(thiophen-2-yl)pent-4-ynamide (16).** Yield 95%, as a yellow greenish oil; IR (NaCl) cm^{-1}: 1679; ^{1}H NMR (250 MHz, $CDCl_3$) δ 9.47 (s, 1H), 8.16 (dd, J = 8.2 Hz, J' = 1.6 Hz, 2H), 7.53-7.63 (m, 3H), 7.13 (d, J = 5.1 Hz, 1H), 7.08 (d, J = 3.8 Hz, 1H), 6.89 (dd, J = 5.2 Hz, J' = 3.8 Hz, 1H), 5.91-6.06 (m, 1H), 5.26 (dd, J = 17.3 Hz, J' = 1.2 Hz, 1H), 5.13 (dd, J = 10.3 Hz, J' = 1.2 Hz, 1H), 4.89 (d, J = 5.3 Hz, 2H), 3.2 (t, J = 7.5 Hz, 2H), 2.88 (t, J = 7.5 Hz, 2H); ^{13}C NMR (62.5 MHz, $CDCl_3$) δ 172.8 (C), 161.4 (C), 156.0 (C), 142.2 (CH), 133.1 (CH), 133.1 (CH), 133.0 (C), 131.3 (CH), 129.6 (2CH), 127.9 (2CH), 126.8 (CH), 126.2 (CH), 123.8 (C), 117.5 (CH_2), 92.9 (C), 74.3 (C), 48.5 (CH_2), 36.9 (CH_2), 16.4 (CH_2); *m/z* (M+1) = 375.

***N*-Allyl-*N*-(5-phenyl-1,2,4-triazin-3-yl)-5-(4-methoxyphenyl)pent-4-ynamide (17).** Yield 89%, as a maroon oil; IR (NaCl) cm^{-1}: 1679; ^1H NMR (250 MHz, $CDCl_3$) δ 9.48 (s, 1H), 8.17 (dd, *J* = 7.8 Hz, *J'* = 1.3 Hz, 2H), 7.54-7.64 (m, 3H), 7.27 (dd, *J* = 6.9 Hz, *J'* = 1.9 Hz, 2H), 6.77 (dd, *J* = 6.9 Hz, *J'* = 1.9 Hz, 2H), 5.91-6.07 (m, 1H), 5.27 (dd, *J* = 17.3 Hz, *J'* = 1.3 Hz, 1H), 5.14 (dd, *J* = 10.0 Hz, *J'* = 1.3 Hz, 1H), 4.89 (d, *J* = 5.3 Hz, 2H), 3.78 (s, 3H), 3.27 (t, *J* = 7.5 Hz, 2H), 2.86 (t, *J* = 7.5 Hz, 2H); ^{13}C NMR (62.5 MHz, $CDCl_3$) δ 173.1 (C), 161.5 (C), 159.2 (C), 156.0 (C), 142.1 (CH), 133.2 (CH), 133.1 (CH), 133.0 (2CH), 129.6 (2CH), 127.9 (2CH), 117.4 (CH_2), 115.9 (C), 113.9 (2CH), 87.2 (C), 80.9 (C), 55.3 (CH_3), 48.5 (CH_2), 37.1 (CH_2), 16.3 (CH_2); *m/z* (M+1) = 399.

***N*-Isopropyl-*N*-(5-phenyl-1,2,4-triazin-3-yl)-5-(thiophen-2-yl)pent-4-ynamide (18).** Yield 91%, as a maroon solid; mp 88-90°C; IR (KBr) cm^{-1}: 1669; ^1H NMR (250 MHz, $CDCl_3$) δ 9.56 (s, 1H), 8.19 (dd, *J* = 8.2 Hz, *J'* = 1.9 Hz, 2H), 7.56-7.65 (m, 3H), 7.12 (dd, *J* = 5.3 Hz, *J'* = 1.3 Hz, 1H), 7.05 (dd, *J* = 3.8 Hz, *J'* = 1.3 Hz, 1H), 6.88 (dd, *J* = 5.3 Hz, *J'* = 3.8 Hz, 1H), 5.08-5.20 (m, 1H), 2.80-2.86 (m, 2H), 2.68-2.75 (m, 2H), 1.39 (d, *J* = 6.9 Hz, 6H); ^{13}C NMR (62.5 MHz, $CDCl_3$) δ 171.5 (C), 161.7 (C), 156.5 (C), 143.5 (CH), 133.3 (CH), 132.96 (C), 131.3 (CH), 129.7 (2CH), 127.9 (2CH), 126.8 (CH), 126.2 (CH), 123.8 (C), 92.9 (C), 74.2 (C), 49.4 (CH), 35.6 (CH_2), 21.0 ($2CH_3$), 16.3 (CH_2); *m/z* (M+1) = 377.

***N*-Isopropyl-*N*-(5-phenyl-1,2,4-triazin-3-yl)-5-(4-methoxyphenyl)pent-4-ynamide (19).** Yield 85%, as a maroon solid; mp 84-86°C; IR (KBr) cm^{-1}: 1661; ^1H NMR (250 MHz, $CDCl_3$) δ 9.59 (s, 1H), 8.18 (d, *J* = 6.6 Hz, 2H), 7.54-7.65 (m, 3H), 7.24 (d, *J* = 8.8 Hz, 2H), 6.74 (d, *J* = 8.8 Hz, 2H), 5.09-5.19 (m, 1H), 3.76 (s, 3H), 2.70-2.79 (m, 2H), 1.39 (d, *J* = 6.9 Hz, 6H); ^{13}C NMR (62.5 MHz, $CDCl_3$) δ 171.7 (C), 161.8 (C), 159.2 (C), 156.4 (C), 143.5 (CH), 133.3 (CH), 133.0 (2CH), 132.8 (C), 129.7 (2CH), 127.9 (2CH), 115.9 (C), 113.8 (2CH), 87.2 (C), 80.8 (C), 55.3 (CH_3), 49.3 (CH), 35.9 (CH_2), 21.1 ($2CH_3$), 16.2 (CH_2); *m/z* (M+1) = 401.

***N*-Phenyl-*N*-(5-phenyl-1,2,4-triazin-3-yl)-5-(thiophen-2-yl)pent-4-ynamide (20).** Yield 86%, as a yellow oil; IR (NaCl) cm^{-1}: 1683; ^1H NMR (250 MHz, $CDCl_3$) δ 9.50 (s, 1H), 8.07 (dd, *J* = 8.0 Hz, *J'* = 1.6 Hz, 2H), 7.38-7.60 (m, 8H), 7.15 (d, *J* = 5.1 Hz, 1H), 7.11 (d, *J* = 3.8 Hz, 1H), 6.91 (dd, *J* = 5.0 Hz, *J'* = 3.8 Hz, 1H), 3.00-3.07 (m, 2H), 2.87-2.94 (m, 2H); ^{13}C NMR (62.5 MHz, $CDCl_3$) δ 172.6 (C), 162.2 (C), 156.6 (C), 142.9 (CH), 140.2 (C), 133.1 (CH), 132.8 (C), 131.4 (CH), 129.7 (2CH), 129.6 (2CH), 129.0 (2CH), 128.5 (CH), 127.9 (2CH), 126.9 (CH), 126.3 (CH), 123.8 (C), 92.7 (C), 74.5 (C), 35.7 (CH_2), 16.2 (CH_2); *m/z* (M+1) = 411.

***N*-Phenyl-*N*-(5-phenyl-1,2,4-triazin-3-yl)-5-(4-methoxyphenyl)pent-4-ynamide (21).** Yield 82%, as a yellow solid; mp 120-122°C; IR (KBr) cm^{-1}: 1692; ^{1}H NMR (250 MHz, $CDCl_3$) δ 9.50 (s, 1H), 8.05 (d, *J* = 7.2 Hz, 2H), 7.58-7.68 (m, 8H), 7.30 (d, *J* = 8.5 Hz, 2H), 6.77 (d, *J* = 8.5 Hz, 2H), 3.77 (s, 3H), 3.03 (t, *J* = 6.9 Hz, 2H), 2.86 (t, *J* = 6.9 Hz, 2H); ^{13}C NMR (62.5 MHz, $CDCl_3$) δ 173.0 (C), 162.2 (C), 159.2 (C), 156.6 (C), 142.9 (CH), 140.3 (C), 133.0 (2CH), 132.8 (C), 129.7 (2CH), 129.5 (2CH), 129.0 (2CH), 128.4 (CH), 127.9 (2CH), 115.8 (C), 113.9 (2CH), 87.0 (C), 81.0 (C), 55.3 (CH_3), 36.0 (CH_2), 16.0 (CH_2); *m/z* (M+1) = 435.

General procedure for the intramolecular inverse electron-demand Diels–Alder reaction

Triazines **6–9** or **14–21** (0.30 mmol) were dissolved in chlorobenzene (2 mL) and heated at 220 °C under microwave irradiation for 1 h. The reaction was purified by chromatography using PE/EtOAc as eluant to give the desired products **10–13** or **22–29**.

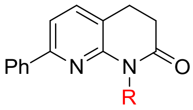

10 - 13

1-Butyl-7-phenyl-3,4-dihydro-1,8-naphthyridin-2(1*H*)-one (10). Yield 97%, as a white solid; mp 74-76°C, after purification by column chromatography (PE-EtOAc, 8.5:1.5); IR (KBr) cm^{-1}: 1662; ^{1}H NMR (250 MHz, $CDCl_3$) δ 8.03 (dd, *J* = 8.5 Hz, *J'* = 1.6 Hz, 2H), 7.69 (m, 5H), 4.27 (t, *J* = 7.4 Hz, 2H), 2.90 (dd, *J* = 8.5 Hz, *J'* = 6.2 Hz, 2H), 2.71 (dd, *J* = 8.5 Hz, *J'* = 6.2 Hz, 2H), 1.67-1.79 (m, 2H), 1.39-1.48 (m, 2H), 0.98 (t, *J* = 7.2 Hz, 3H); ^{13}C NMR (62.5 MHz, $CDCl_3$) δ 170.7 (C), 153.8 (C), 151.7 (C), 138.9 (C), 136.5 (CH), 129.0 (CH), 128.8 (2CH), 126.6 (2CH), 119.2 (C), 114.2 (CH), 40.7 (CH_2), 31.6 (CH_2), 30.1 (CH_2), 23.8 (CH_2), 20.5 (CH_2), 14.1 (CH_3); *m/z* (M+1) = 281. Anal. Calcd for $C_{18}H_{20}N_2O$: C, 77.11; H, 7.19; N, 9.99. Found: C, 77.17; H, 7.12; N, 10.06.

1-Allyl-7-phenyl-3,4-dihydro-1,8-naphthyridin-2(1*H*)-one (11). Yield 96%, as a white solid; mp 88-90°C, after purification by column chromatography (PE-EtOAc, 8.5:1.5); IR (KBr) cm^{-1}: 1670; ^{1}H NMR (250 MHz, $CDCl_3$) δ 8.02 (dd, *J* = 6.9 Hz, *J'* = 1.9 Hz, 2H), 7.38-7.52 (m, 5H), 5.95-6.12 (m, 1H), 5.28 (dd, *J* = 17.3 Hz, *J'* = 1.6 Hz, 1H), 5.15 (dd, *J* = 10.4 Hz, *J'* = 1.6 Hz, 1H), 4.90 (d, *J* = 4.2 Hz, 2H), 2.93

(dd, J = 8.8 Hz, J' = 6.0 Hz, 2H), 2.75 (dd, J = 8.8 Hz, J' = 6.0 Hz, 2H); ^{13}C NMR (62.5 MHz, $CDCl_3$) δ 171.5 (C), 153.9 (C), 151.4 (C), 138.8 (C), 136.6 (CH), 133.6 (CH), 129.0 (CH), 128.8 (2CH), 126.6 (2CH), 119.0 (C), 116.7 (CH_2), 114.5 (CH), 43.0 (CH_2), 31.6 (CH_2), 23.7 (CH_2); *m/z* (M+1) = 265. Anal. Calcd for $C_{17}H_{16}N_2O$: C, 77.25; H, 6.10; N, 10.60. Found: C, 77.18; H, 6.17; N, 10.69.

1-Isopropyl-7-phenyl-3,4-dihydro-1,8-naphthyridin-2(1*H*)-one (12). Yield 93%, as a yellow solid; mp 112-114°C, after purification by column chromatography (PE-EtOAc, 8.5:1.5); IR (KBr) cm^{-1}: 1665; ^{1}H NMR (250 MHz, $CDCl_3$) δ 8.02 (dd, J = 6.6 Hz, J' = 1.4 Hz, 2H), 7.37-7.50 (m, 5H), 5.35-5.47 (m, 1H), 2.85 (dd, J = 8.2 Hz, J' = 5.6 Hz, 2H), 2.66 (dd, J = 8.2 Hz, J' = 5.6 Hz, 2H), 1.61 (d, J = 6.9 Hz, 6H); ^{13}C NMR (62.5 MHz, $CDCl_3$) δ 171.4 (C), 153.6 (C), 152.5 (C), 139.0 (C), 136.3 (CH), 129.0 (CH), 128.8 (2CH), 126.6 (2CH), 120.5 (C), 114.4 (CH), 46.0 (CH), 32.8 (CH_2), 24.1 (CH_2), 20.2 ($2CH_3$); *m/z* (M+1) = 267.

1,7-Diphenyl-3,4-dihydro-1,8-naphthyridin-2(1*H*)-one (13). Yield 98%, as a yellow solid; mp 196-198°C, after purification by column chromatography (PE-EtOAc, 6:4); IR (KBr) cm^{-1}: 1688; ^{1}H NMR (250 MHz, $CDCl_3$) δ 7.67 (dd, J = 6.8 Hz, J' = 2.2 Hz, 2H), 7.39-7.58 (m, 5H), 7.27-7.30 (m, 5H), 3.08 (dd, J = 8.5 Hz, J' = 6.0 Hz, 2H), 2.91 (dd, J = 8.5 Hz, J' = 6.0 Hz, 2H); ^{13}C NMR (62.5 MHz, $CDCl_3$) δ 170.9 (C), 153.7 (C), 152.9 (C), 138.3 (C), 137.7 (C), 136.7 (CH), 129.4 (2CH), 129.1 (2CH), 129.0 (CH), 128.7 (2CH), 127.8 (CH), 126.4 (2CH), 118.8 (C), 114.5 (CH), 32.1 (CH_2), 23.9 (CH_2); *m/z* (M+1) = 301.

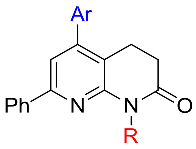

22 - 29

1-Butyl-7-phenyl-5-(thiophen-2-yl)-3,4-dihydro-1,8-naphthyridin-2(1*H*)-one (22). Yield 74%, as a maroon solid; mp 130-132°C, after purification by column chromatography (PE-EtOAc, 8:2); IR (KBr) cm^{-1}: 1679; ^{1}H NMR (250 MHz, $CDCl_3$) δ 8.05 (dd, J = 8.2 Hz, J' = 1.5 Hz, 2H), 7.44-7.52 (m, 5H), 7.17 (d, J = 3.8 Hz, 2H), 4.31 (t, J = 7.5 Hz, 2H), 3.06 (dd, J = 8.5 Hz, J' = 7.2 Hz, 2H), 2.65 (dd, J = 8.5 Hz, J' = 7.2 Hz, 2H), 1.70-1.83 (m, 2H), 1.39-1.54 (m, 2H), 1.00 (t, J = 7.2 Hz, 3H); ^{13}C NMR (62.5 MHz, $CDCl_3$) δ 170.6 (C), 153.4 (C), 152.6 (C), 142.2 (C), 139.6 (C), 138.6 (C), 129.2 (CH), 128.8 (2CH), 128.1 (CH), 127.8 (CH), 127.1 (CH), 126.6 (2CH), 117.0 (C), 116.0 (CH), 41.2 (CH_2), 31.5 (CH_2), 30.2 (CH_2), 21.8

(CH_2), 20.5 (CH_2), 14.1 (CH_3); *m/z* (M+1) = 363. Anal. Calcd for $C_{22}H_{22}N_2OS$: C, 72.90; H, 6.12; N, 7.73. Found: C, 72.82; H, 6.19; N, 7.68.

1-Butyl-5-(4-methoxyphenyl)-7-phenyl-3,4-dihydro-1,8-naphthyridin-2(1*H*)-one (23). Yield 80%, as a maroon solid; mp 120-122°C, after purification by column chromatography (PE-EtOAc, 8:2); IR (KBr) cm^{-1}: 1674; ^1H NMR (250 MHz, $CDCl_3$) δ 8.06 (dd, *J* = 8.5 Hz, *J'* = 1.6 Hz, 2H), 7.38-7.51 (m, 4H), 7.31 (d, *J* = 8.8 Hz, 2H), 7.02 (d, *J* = 8.8 Hz, 2H), 4.32 (t, *J* = 7.5 Hz, 2H), 3.88 (s, 3H), 2.89 (dd, *J* = 8.5 Hz, *J'* = 6.2 Hz, 2H), 2.61 (dd, *J* = 8.5 Hz, *J'* = 6.2 Hz, 2H), 1.72-1.85 (m, 2H), 1.40-1.56 (m, 2H), 1.00 (t, *J* = 7.2 Hz, 3H); ^{13}C NMR (62.5 MHz, $CDCl_3$) δ 170.8 (C), 159.8 (C), 153.2 (C), 152.3 (C), 149.4 (C), 138.9 (C), 130.9 (C), 130.0 (2CH), 129.0 (CH), 128.8 (2CH), 126.6 (2CH), 117.0 (C), 116.0 (CH), 114.1 (2CH), 55.5 (CH_3), 41.1 (CH_2), 31.7 (CH_2), 30.2 (CH_2), 21.6 (CH_2), 20.5 (CH_2), 14.1 (CH_3); *m/z* (M+1) = 387. Anal. Calcd for $C_{25}H_{26}N_2O_2$: C, 77.69; H, 6.78; N, 7.25. Found: C, 77.76; H, 6.81; N, 7.19.

1-Allyl-7-phenyl-5-(thiophen-2-yl)-3,4-dihydro-1,8-naphthyridin-2(1*H*)-one (24). Yield 73%, as a yellow oil, after purification by column chromatography (PE-EtOAc, 8:2); IR (NaCl) cm^{-1}: 1697; ^1H NMR (250 MHz, $CDCl_3$) δ 8.03 (dd, *J* = 8.2 Hz, *J'* = 1.5 Hz, 2H), 7.40-7.52 (m, 5H), 7.17 (dd, *J* = 4.0 Hz, *J'* = 1.4 Hz, 2H), 5.97-6.14 (m, 1H), 5.30 (dd, *J* = 17.3 Hz, *J'* = 1.5 Hz, 1H), 5.17 (dd, *J* = 10.3 Hz, *J'* = 1.5 Hz, 1H), 4.90 (d, *J* = 5.4 Hz, 2H), 3.08 (dd, *J* = 8.5 Hz, *J'* = 6.0 Hz, 2H), 2.68 (dd, *J* = 8.5 Hz, *J'* = 6.0 Hz, 2H); ^{13}C NMR (62.5 MHz, $CDCl_3$) δ 170.4 (C), 153.5 (C), 152.3 (C), 142.4 (C), 139.5 (C), 138.5 (C), 133.7 (CH), 129.2 (CH), 128.9 (2CH), 128.1 (CH), 127.8 (CH), 127.1 (CH), 126.7 (2CH), 116.8 (C), 116.8 (CH_2), 116.2 (CH), 43.5 (CH_2), 31.4 (CH_2), 21.7 (CH_2); *m/z* (M+1) = 347.

1-Allyl-5-(4-methoxyphenyl)-7-phenyl-3,4-dihydro-1,8-naphthyridin-2(1*H*)-one (25). Yield 79%, as a yellow solid; mp 96-98°C, after purification by column chromatography (PE-EtOAc, 8:2); IR (KBr) cm^{-1}: 1679; ^1H NMR (250 MHz, $CDCl_3$) δ 8.02 (d, *J* = 7.4 Hz, 2H), 7.40-7.50 (m, 4H), 7.31 (dd, *J* = 6.6 Hz, *J'* = 1.9 Hz, 2H), 7.02 (d, *J* = 8.5 Hz, 2H), 6.00-6.17 (m, 1H), 5.32 (dd, *J* = 15.7 Hz, *J'* = 1.6 Hz, 1H), 5.18 (dd, *J* = 10.3 Hz, *J'* = 1.6 Hz, 1H), 4.95 (d, *J* = 5.6 Hz, 2H), 3.88 (s, 3H), 2.92 (dd, *J* = 8.8 Hz, *J'* = 6.0 Hz, 2H), 2.65 (dd, *J* = 8.8 Hz, *J'* = 6.0 Hz, 2H); ^{13}C NMR (62.5 MHz, $CDCl_3$) δ 170.7 (C), 159.8 (C), 153.3 (C), 152.0 (C), 149.6 (C), 138.8 (C), 133.8 (CH), 130.8 (C), 130.0 (2CH), 129.1 (CH), 128.8 (2CH), 126.7 (2CH), 116.9 (C), 116.7 (CH_2), 116.3 (CH), 114.1 (2CH), 55.5 (CH_3), 43.4 (CH_2), 21.6 (CH_2), 31.6 (CH_2); *m/z* (M+1) = 371.

1-Isopropyl-7-phenyl-5-(thiophen-2-yl)-3,4-dihydro-1,8-naphthyridin-2(1*H*)-one (26). Yield 91%, as a yellow solid; mp 136-138°C, after purification by column chromatography (PE-EtOAc, 8:2); IR (KBr) cm^{-1}: 1674; ^1H NMR (250 MHz, $CDCl_3$) δ 8.06 (dd, *J* = 8.0 Hz, *J'* = 1.4 Hz, 2H), 7.42-7.52 (m, 5H), 7.17 (d, *J* = 3.4 Hz, 2H), 5.34-5.48 (m, 1H), 3.02 (dd, *J* = 8.5 Hz, *J'* = 6.9 Hz, 2H), 2.60 (dd, *J* = 8.5 Hz, *J'* = 6.9 Hz, 2H), 1.64 (d, *J* = 6.9 Hz, 6H); ^{13}C NMR (62.5 MHz, $CDCl_3$) δ 171.3 (C), 153.4 (C), 153.2 (C), 142.1 (C), 139.7 (C), 138.7 (C), 129.2 (CH), 128.9 (2CH), 128.1 (CH), 127.8 (CH), 127.1 (CH), 126.7 (2CH), 118.4 (C), 116.1 (CH), 46.6 (CH), 32.6 (CH_2), 22.0 (CH_2), 20.3 ($2CH_3$); *m/z* (M+1) = 349.

1-Isopropyl-5-(4-methoxyphenyl)-7-phenyl-3,4-dihydro-1,8-naphthyridin-2(1*H*)-one (27). Yield 94%, as a yellow solid; mp 102-104°C, after purification by column chromatography (PE-EtOAc, 8:2); IR (KBr) cm^{-1}: 1679; ^1H NMR (250 MHz, $CDCl_3$) δ 8.06 (dd, *J* = 6.6 Hz, *J'* = 1.3 Hz, 2H), 7.40-7.52 (m, 4H), 7.32 (dd, *J* = 6.6 Hz, *J'* = 1.9 Hz, 2H), 7.02 (dd, *J* = 6.6 Hz, *J'* = 1.9 Hz, 2H), 5.37-5.49 (m, 1H), 3.88 (s, 3H), 2.85 (dd, *J* = 9.4 Hz, *J'* = 6.6 Hz, 2H), 2.56 (dd, *J* = 9.4 Hz, *J'* = 6.6 Hz, 2H), 1.65 (d, *J* = 6.9 Hz, 6H); ^{13}C NMR (62.5 MHz, $CDCl_3$) δ 171.6 (C), 159.8 (C), 153.1 (C), 153.0 (C), 149.3 (C), 139.0 (C), 131.0 (C), 130.1 (2CH), 129.0 (CH), 128.8 (2CH), 126.7 (2CH), 118.4 (C), 116.1 (CH), 114.1 (2CH), 55.5 (CH_3), 46.5 (CH), 32.8 (CH_2), 21.9 (CH_2), 20.3 ($2CH_3$); *m/z* (M+1) = 373.

1,7-Diphenyl-5-(thiophen-2-yl)-3,4-dihydro-1,8-naphthyridin-2(1*H*)-one (28). Yield 78%, as a maroon solid; mp 196-198°C, after purification by column chromatography (PE-EtOAc, 8:2); IR (KBr) cm^{-1}: 1693; ^1H NMR (250 MHz, $CDCl_3$) δ 7.71 (dd, *J* = 7.2 Hz, *J'* = 2.5 Hz, 2H), 7.40-7.52 (m, 5H), 7.30-7.35 (m, 5H), 7.18-7.27 (m, 2H), 3.25 (dd, *J* = 8.5 Hz, *J'* = 6.4 Hz, 2H), 2.87 (dd, *J* = 8.5 Hz, *J'* = 6.4 Hz, 2H); ^{13}C NMR (62.5 MHz, $CDCl_3$) δ 170.9 (C), 153.9 (C), 153.3 (C), 142.5 (C), 139.5 (C), 138.1 (C), 138.0 (C), 129.4 (2CH), 129.2 (CH), 129.1 (2CH), 128.7 (2CH), 128.2 (CH), 127.9 (CH), 127.8 (CH), 127.2 (CH), 126.5 (2CH), 116.6 (C), 116.1 (CH), 32.0 (CH_2), 22.0 (CH_2); *m/z* (M+1) = 383.

1,7-Diphenyl-5-(4-methoxyphenyl)-3,4-dihydro-1,8-naphthyridin-2(1*H*)-one (29). Yield 84%, as a yellow greenish solid; mp 240-242°C, after purification by column chromatography (PE-EtOAc, 8:2); IR (KBr) cm^{-1}: 1693; ^1H NMR (250 MHz, $CDCl_3$) δ 7.70 (dd, *J* = 6.6 Hz, *J'* = 2.8 Hz, 2H), 7.24-7.56 (m, 11H), 7.04 (d, *J* = 8.4 Hz, 2H), 3.06 (s, 3H), 3.06 (dd, *J* = 8.6 Hz, *J'* = 6.0 Hz, 2H), 2.81 (dd, *J* = 8.6 Hz, *J'* = 6.0 Hz, 2H); ^{13}C NMR (62.5 MHz, $CDCl_3$) δ 171.1 (C), 159.9 (C), 153.6 (C), 153.1 (C), 149.8 (C), 138.3 (C), 138.2 (C), 130.8 (C), 130.0 (2CH), 129.4 (2CH), 129.1 (2CH), 129.0 (CH), 128.7 (2CH), 127.8 (CH), 126.4 (2CH), 116.7 (C), 116.2 (CH), 114.2 (2CH), 55.6 (CH_3), 32.2 (CH_2), 21.8 (CH_2); *m/z* (M+1) = 256.
